# In situ produced exopolysaccharides by *Bacillus coagulans* IBRC‐M 10807 and its effects on properties of whole wheat sourdough

**DOI:** 10.1002/fsn3.3624

**Published:** 2023-09-07

**Authors:** Zahra Farajinejad, Forogh Mohtarami, Mirkhalil Pirouzifard, Saber Amiri, Hamed Hamishehkar

**Affiliations:** ^1^ Department of Food Science and Technology, Faculty of Agriculture Urmia University Urmia Iran; ^2^ Drug Applied Research Center Tabriz University of Medical Sciences Tabriz Iran

**Keywords:** fermentation, Fructooligosaccharides, prebiotic, spore‐forming probiotic

## Abstract

This study aimed to investigate in situ exopolysaccharides (EPSs) production by *Bacillus coagulans* IBRC‐M 10807 under different fermentation conditions to improve the technical‐functional properties of whole wheat flour sourdough and obtain high‐quality products. For this purpose, the effectiveness of four efficient factors including *B. coagulans* (8 Log CFU/g), FOS (0%, 2.5%, and 5% based on flour weight), fermentation temperature (30, 35, and 40°C), and fermentation time (12, 18, and 24 h) was investigated on the production of functional sourdough. Our work focused on optimizing probiotic sourdough by investigating probiotic viability, pH, total titratable acidity, antioxidant properties, and EPS measurement. The first optimal formulation for maximized production of the in situ EPSs by the numerical optimization included FOS 0%, *B. coagulans* IBRC‐M 10807 8 Log CFU/g, fermentation temperature of 30°C, and fermentation time of 12 h. In this case, EPSs was 59.28 mg/g and probiotic was 10.99 Log CFU/g. The second optimal formula by considering the highest viability of probiotic together with EPS production was determined as FOS 4.71%, *B. coagulans* IBRC‐M 10807, 8 Log CFU/g, fermentation temperature of 30°C, and fermentation time of 20 h. The predicted amount of the EPSs and probiotic viability via the second formulation were 54.4 mg/g and 11.18 Log CFU/g, respectively. Analyses of optimal sourdough using FTIR, SEM, and DSC revealed that FOS and probiotics significantly reduced the enthalpy of amylopectin retrogradation and delayed it compared to other samples. Therefore, improving the final product's technological capabilities and shelf life can be credited with potential benefits.

## Graphical abstract

The effects of *B. coagulans*, FOS (Fructooligosaccharides), fermentation temperature, and fermentation time were investigated on the production of functional sourdough. The longer the fermentation time and higher fermentation temperatures, the less in‐situ EPS production. The antistaling ability of FOS with probiotics was confirmed by DSC, FTIR, and SEM.

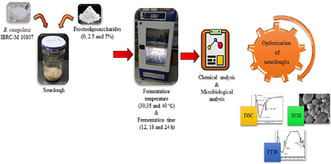

## 1 INTRODUCTION

Whole wheat flour is an excellent exogenous source of amino acids, dietary fiber, trace elements, carbohydrates, and vitamins (Sakandar et al., 2018). Over the past few decades, consumer demand for fresh and minimally processed foods without additional chemical preservatives has continued to grow (Gaglio et al., 2020; Nionelli et al., 2018; Preedy & Watson, 2019). Sourdough comprises a highly complex, nonaseptic fermentation ecosystem that can be utilized as a generally recognized safe (GRAS) biological additive in bread making (Abedfar & Sadeghi, 2019; Sadeghi et al., 2019). The most significant factors affecting sourdough fermentation are microbial diversity, fermentation conditions (yield sourdough, temperature, time, and number of back‐slopping), and flour composition (especially carbohydrates, protein, and ash contents) (Abedfar & Sadeghi, 2019; Sadeghi et al., 2019). The incorporation of sourdough into bread processing has demonstrated significant technical, nutritional, and economic effects (Catzeddu, 2019). Its positive effects include creating a softer crumb structure, delaying the onset of microbial spoilage, and increasing nutritional properties (Lynch et al., 2018). These positive effects of sourdough and fermentation are mainly related to acidification and changes in pH, which can directly affect the structural components of the dough and the activity of endogenous enzymes in cereals (Karimi et al., 2021; Lynch et al., 2018; Preedy & Watson, 2019). High acidity hurts the dough and can be corrected using lactic acid bacteria (LAB) exopolysaccharides (EPSs) (Lynch et al., 2018). EPSs are a potential natural alternative to commercial hydrocolloids, including hydroxypropyl methylcellulose (HPMC) (Galli et al., 2020; Lynch et al., 2018). In this regard, in the cereal and bakery industry, the application of an EPS‐producing medium has been considered (Galli et al., 2020; Lynch et al., 2018). Numerous studies such as Dertli et al. (2016), İspirli et al. (2020), Galli et al. (2020), Zhang et al. (2021), and Bockwoldt et al. (2021) have been performed on the in situ EPS production in the sourdough. The positive effects of EPS include increasing the water absorption of dough, increasing the volume of bread, increasing the rheology of dough, maintaining the structure of bread, increasing the hardness of the crumb, and delaying the bread staling, which therefore increases the shelf life of bread (Lynch et al., 2018). Since LAB made away with baking, recent research has shown that lactic acid‐producing bacteria, especially bacilli, remain stable in cooked foods and retain their probiotic benefits at cooking temperatures (Dolin, 2009). *Bacillus coagulans* IBRC‐M 10807 (*B. coagulans)* is a species of *bacillus* that could be used due to its inherent property (resistance to harsh conditions by spore formation) in the cooking process (Sudha et al., 2010).

*B. coagulans* is a Gram‐positive, lactic acid‐producing, rod‐shaped, spore‐forming, aerobic, or facultative anaerobic bacterium within the genus *Bacillus* (Sui et al., 2020). Its optimum growth temperature is 35–50°C, and its optimum pH is 5.5–6.5 (Cao et al., 2020). During growth, *B. coagulans* can break down glucose, maltose, sucrose, and mannitol and produce lactic acid, which shows the properties of *Lactobacillus* and *Bacillus*. It also has unique characteristics such as probiotic properties, high‐temperature resistance, and easy storage (Asianezhad et al., 2023).

Over the past few years, increasing awareness of health has led people to pay more attention to their eating habits. Since functional foods, in addition to meeting nutritional needs, play a vital role in maintaining the health of the human body, much attention has been paid to them (Singh et al., 2016). Prebiotics make up the majority of functional foods, which are indigestible foods affecting the host's health by selectively stimulating the growth and/or activity of one or a limited number of bacteria in the large intestine. FOS are GRAS and are used as functional foods due to their prebiotic properties, in addition to their economic potential and beneficial properties (de la Rosa et al., 2019; Singh et al., 2016).

As far as we know, this is the first study on the effect of FOS, *B. coagulans* IBRC‐M 10807, and the simultaneous impact of FOS and *B. coagulans* on whole wheat sourdough fermentation. To enhance the technical and rheological qualities of whole wheat sourdough, this study evaluated the impact of in situ produced EPSs by *B. coagulans* in type II sourdough (free yeast). It also determined the optimal amount of FOS (as a prebiotic) and the optimum temperature and time of sourdough fermentation.

## 2 MATERIALS AND METHODS

### 2.1 Materials

Lyophilized *B. coagulans* IBRC‐M 10807 was purchased from the Iranian Biological Research Center (IBRC). FOS was obtained from Sigma‐Aldrich. The whole wheat flour was bought from a local milling factory (Athar Flour) and chemically analyzed according to the AACC's standard methods and contained moisture, protein, carbohydrates, fat, and ash value of whole wheat flour was 11.31%, 12.04%, 72.65%, 2.42%, and 1.58%, respectively. The sucrose was purchased from a local market. The microbial media including de Man, Rogosa, and Sharpe (MRS) broth, MRS agar, and Nutrient Yeast Extract Salt Medium (NYSM) broth and all other chemicals were bought from Merck.

### 2.2 Methods

#### 2.2.1 Activation of *B. coagulans* and growth conditions

Freeze‐dried *B. coagulans* cells were cultivated in MRS broth at 37°C for 48 h. After culture, biomass was collected with cold centrifugation (Sigma 2‐16KL, Sigma) for 15 min at 5000× *g* (4°C) and washed twice in sterile normal saline, and prepared to add 8 Log CFU/g (Mokarram et al., 2009; Sadeghi et al., 2019).

#### 2.2.2 Preparation of sourdough

According to preliminary studies, to prepare the control sourdough sample, first whole wheat flour and sterile distilled water were mixed at a ratio of 1:1 (obtaining a sourdough yield of 200 (sourdough weight × 100/flour weight)) in glass containers with sterile lids in sterile conditions. Sucrose in all treatments was substituted with 2% (w/w) of flour to ensure EPS production. In the other treatments, FOS was added at 2.5% and 5% (based on flour weight) to the mixture inoculated with *B. coagulans* suspension (8 Log CFU/g). Finally, all starters of 17 runs (Table 1) in sealed containers were fermented in incubators at different fermentation temperatures (30, 35, and 40°C) and times (12, 18, and 24 h) (İspirli et al., 2020; Siepmann et al., 2019).

**TABLE 1:** The matrix of Box–Behnken design for sourdough production.

Run	Factor 1	Factor 2	Factor 3
	FOS (% W/W)	Fermentation temperature (°C)	Fermentation time (h)
1	0	30	18
2	2.5	35	18
3	5	40	18
4	0	35	12
5	5	35	12
6	2.5	40	12
7	5	35	24
8	2.5	35	18
9	2.5	30	24
10	0	40	18
11	5	30	18
12	2.5	40	24
13	0	35	24
14	2.5	35	18
15	2.5	35	18
16	2.5	35	18
17	2.5	30	12

Abbreviation: FOS, fructooligosaccharides.

#### 2.2.3 Determination of pH and total titratable acidity

For measuring pH and total titratable acidity (TTA) for each sample, 10 g of sample with 90 mL of distilled water was homogenized. The pH of the samples was determined by a pH meter (Metrohm 827) which was calibrated using 7 pH and 4 pH buffers, respectively. Then, the samples were titrated with 0.1 N NaOH to a final pH of 8.5. TTA was expressed as the volume of NaOH spent in mL (Yildirim & Arici, 2019).

#### 2.2.4 Extraction and estimate of the EPS content

The sourdough samples were diluted 1:2 with distilled water and centrifuged at 11,000× *g* for 10 min. Two volumes of chilled ethanol were added to the supernatants and were stored at 4°C for 24 h in a refrigerator. The precipitate was collected by cold centrifugation at 11,000× *g* for 10 min, and then EPSs were dissolved in deionized water. Two volumes of chilled ethanol were added to the mixture and centrifuged again after about 2 h at 4°C storage. The number of EPSs in each sample was determined by gravimetric methods after freeze‐drying (Christ Alpha 1–4, Braun Biotech International). FOS (3 g) was added to NYSM broth (15 mL) and incubated at 30, 35, and 40°C for 12, 18, and 24 h. FOS consumption by *B. coagulans* was quantified by HPLC (AZURA® HPLC Compact iso, Knauer) using an EyroChem 2000 date control system, an RI detector, and a Shodex NHP column filled with aminopropyl polymer, 67%–33% acetonitrile–water mixture (mobile phase), and a flow rate of 0.80 mL/min at 20°C. Preparation 20% FOS served as the primary standard mixture. The final content of EPSs in the sourdough was calculated by reducing the amount of FOS remaining in the sourdough from the amount of the determined EPSs (Galli et al., 2020; Schwab et al., 2008).

#### 2.2.5 Antioxidant activity of sourdough methanolic extracts

The 2,2‐diphenyl‐1‐picrylhydrazyl (DPPH) radical scavenging activity was determined on the methanolic extract (ME) of the fermented sourdoughs. ME was prepared according to the method of Nionelli et al. (2018) with some modifications. ME was obtained by adding 2.5 g of each sample with 25 mL of 80% methanol. The mixture was centrifuged at 4600× *g* for 20 min after purification by stirring under stream nitrogen for 30 min. ME was transformed into test tubes, purified by nitrogen stream, and kept at 4°C in a refrigerator before assaying antioxidant activity. ME (1 mL) was added to a mixture of 4‐mL methanol and 1 mL of freshly prepared DPPH solution (100 μM) and then incubated in a dark room for 30 min at 25°C. The absorbance was measured spectrophotometrically (Ultrospec 2000 UV/Visible Spectrophotometer, Pharmacia Biotech) at 517 nm and expressed as follows:

$$\text{DPPH scavenging activity}\;\left(\%\right)=\left(\left(\text{blank absorbance}-\text{sample absorbance}\right)/\text{blank absorbance}\right)\times 100$$

#### 2.2.6 Enumeration of *B. coagulans*

Each sourdough sample (10 g) was mixed with 90 mL of sterile normal saline (0.85% NaCl) and homogenized for preparing serial dilutions. Following diluted suspensions were plated on the MRS agar and incubated for 48 h at 37°C to enumerate *B. coagulans* count (Gholam‐Zhiyan et al., 2021; Mokarram et al., 2009).

#### 2.2.7 Scanning electron microscopy (SEM)

Optimal sourdoughs were freeze‐dried and tested for their morphology to observe the potential effect of in situ EPS production by SEM (Philips XL 30 FEG). The samples were covered with a thin layer of gold (DST1, Nanostructured Coating Co.), afterward observed at an accelerating voltage of 5.0 kV with a magnification level of 500, 1500, and 6000× (İspirli et al., 2020).

#### 2.2.8 Fourier transform infrared (FTIR) measurements

FTIR spectra of the sourdough samples were measured using the FTIR spectrometer (Equinox 55LS 101, Bruker). The spectra were obtained in the wavenumber range of 4000–400 cm^−1^ at a 4 cm^−1^ resolution (Siepmann et al., 2019).

#### 2.2.9 Differential scanning calorimetry (DSC) measurement

Differential scanning calorimetry (DSC 200 F3 Maia, Netzsch) equipped with a liquid‐nitrogen intercooler was used for the analysis of thermal properties in sourdough samples. Some of the sourdough samples were placed into the DSC aluminum pans. The sample and empty pans were put in the DSC testing cell and heated from 25 to 250°C with a temperature increase rate of 5°C/min. Using DSC thermograms, the denaturation peak temperature (*T*_p_) and enthalpy (Δ*H*, J/g) were determined (Aprodu et al., 2019).

#### 2.2.10 Statistical design and analysis

In this study, the response surface methodology with the Box–Behnken design with 17 (12 factorial and 5 center points) runs was used to investigate the effect of three independent variables including FOS content (0%, 2.5%, and 5%), fermentation temperature (30, 35, and 40°C), and fermentation time (12, 18, and 24 h) on EPS production in the sourdough during fermentation by *B. coagulans* (Table 1). pH, TTA, EPSs, DPPH, and *B. coagulans enumeration of* sourdough samples were determined. Numerical optimization to maximize EPSs and also probiotic counts was done to predict the optimal condition based on the desirability function. Validation of optimization was done on two optimized samples and their control sample (Table 2). SEM, FTIR, and DSC were performed on the optimal samples. The experimental design, statistical analysis, and numerical optimization were done by Design‐Expert software version 12 (State‐Ease Minneapolis).

**TABLE 2:** Controlled fermentation conditions for optimal sourdoughs and their controls.

Samples	FOS (%)	*B. coagulans* (Log (CFU/g))	Fermentation temperature (°C)	Fermentation time (h)
A	0	1.5 × 10^8^	30	12
B	4.70	1.5 × 10^8^	30	20
C	0	0	30	12
D	0	0	30	20
E	0	1.5 × 10^8^	30	20
F	4.70	0	30	20

Abbreviations: A and B, Optimized samples; C, Control of A (without *B. coagulans*); D, E, and F, Controls of sample B without FOS and *B. coagulans*, without *B. coagulans*, and without FOS, respectively; FOS, fructooligosaccharides.

## 3 RESULTS AND DISCUSSION

### 3.1 Sourdough pH and TTA

According to the results, the single and interaction effects of fermentation time, fermentation temperature, and FOS on the pH and TTA were statistically significant (*p* < .05).

**FIGURE 1:**
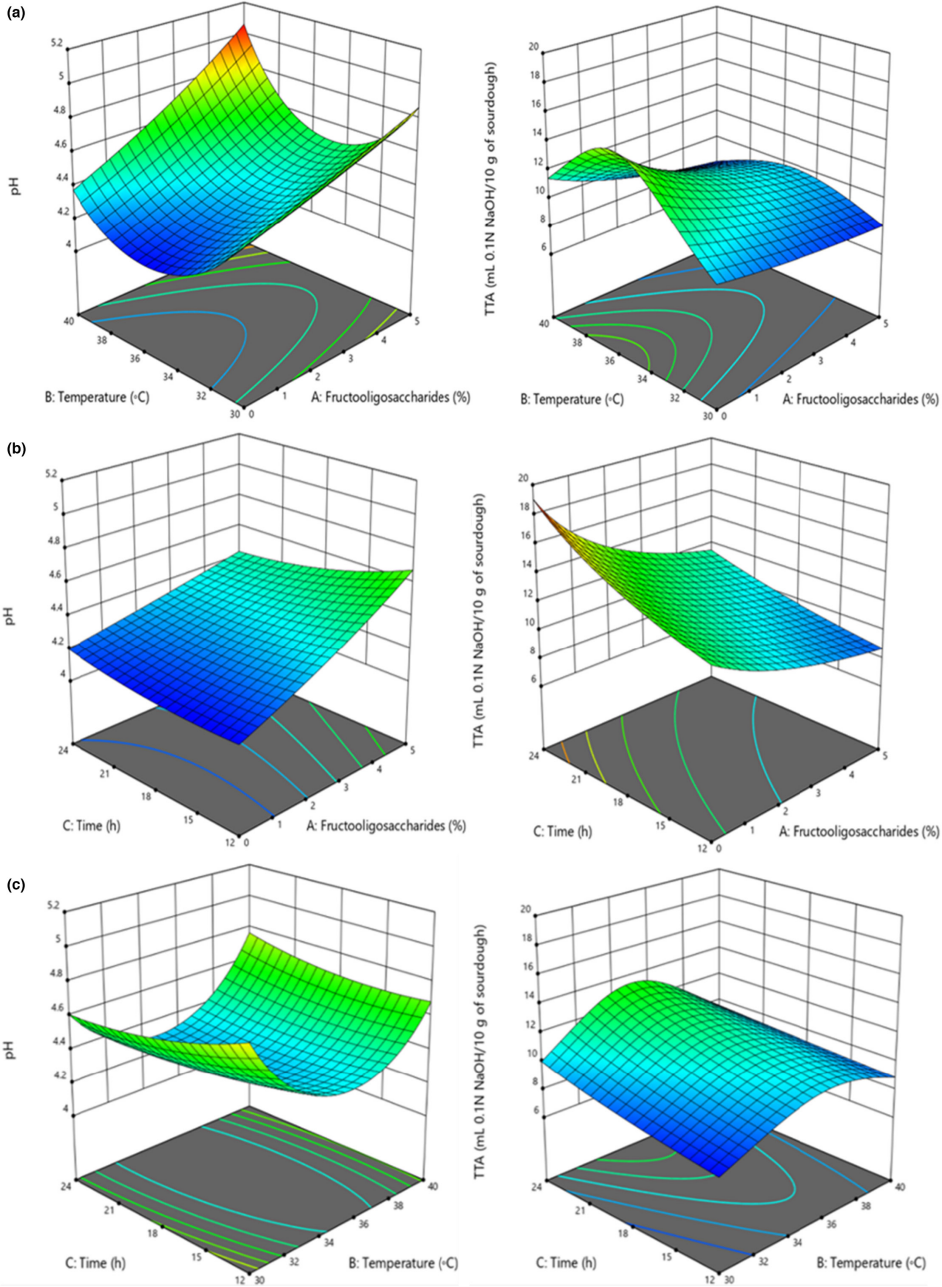
Interaction of (a) FOS content (%) with fermentation temperature (°C), (b) FOS contents (%) with fermentation time (h), and (c) fermentation temperature (°C) with fermentation time (h) on pH and TTA.

As shown in Figure 1a, the effect of fermentation temperature on pH and TTA depends on FOS content. Increasing fermentation temperature from 30 to 35°C at high FOS content decreased pH and increased TTA. But fermentation temperature (30–35°C) did not have a noticeable effect on pH and TTA at the low FOS content (*p* > .05). Bacteria in sourdough produce organic acids by consuming sugars in the environment. The presence of prebiotics intensifies this action, leading to a lower pH and higher TTA. But at high temperatures of fermentation (35–40°C) with high contents of FOS, pH increased and TTA decreased. It could be related to the fact that at high temperatures and FOS content, *B. coagulans* probably produced more biomass rather than manufacturing organic acids.

According to Figure 1b, which illustrates the interaction effect of FOS‐fermentation time, the increasing FOS content from 0% to 5%, and fermentation time from 12 to 24 h caused a slight increase in pH and a decrease in TTA, which can be due to the high amount of ash in whole wheat flour. It may be supposed that microbial metabolism, under the influence of fiber buffer capacity and the role of bran as a dietary supplement, alters the pH and TTA profiles under the same fermentation conditions (Abedfar & Sadeghi, 2019).

By increasing the temperature (30–35°C) and time (12–24 h) of the fermentation, pH diminished while TTA increased (Figure 1c). In sourdough, bacteria consume sugars in the environment to produce organic acids. The presence of prebiotics intensifies this action leading to a lower pH and higher TTA. On the other hand, increasing fermentation temperature (35–40°C) increased pH and decreased TTA. Due to the increase in fermentation temperature and contents of FOS, *B. coagulans* produce more mass than organic acids, resulting in an increase in pH and a decrease in TTA.

The analysis of experimental data suited the quadratic mathematical model, which gave the relationship between independent variables as follows:

(1) $$\mathrm{pH}=0.0176-0.0021\;A+0.0003\;C-0.0006\;\mathrm{AB}+0.0008\;\mathrm{AC}-0.0006\;\mathrm{BC}-0.0036\;B^{2}-0.0004\;C^{2}\;\left(R^{2}=0.998;\mathrm{adj}–R^{2}=0.997\right)$$

(2) $$\begin{array}{cc} \mathrm{TTA}\;\left(\mathrm{mL}\;\text{NaOH}/g\;\text{of sourdough}\right)= & 0.0081+0.0031\;A-0.0009\;B\\ & -0.0023\;C+0.0008\;\mathrm{AB}-0.0006\;\mathrm{AC}+0.0014\;\mathrm{BC}-0.0004\;A^{2}\\ & +0.0051\;B^{2}\;\left(R^{2}=0.990;\mathrm{adj}–R^{2}=0.998\right) \end{array}$$

A = FOS content (%), B = Fermentation temperature (°C), and C = Fermentation time (h)

### 3.2 EPS available for sourdoughs

The results showed a statistically significant effect of fermentation time, temperature, and FOS content on the amount of EPSs in the sourdough (*p* < .05). Furthermore, the interactions of fermentation temperature with FOS content significantly affected the EPS production.

**FIGURE 2:**
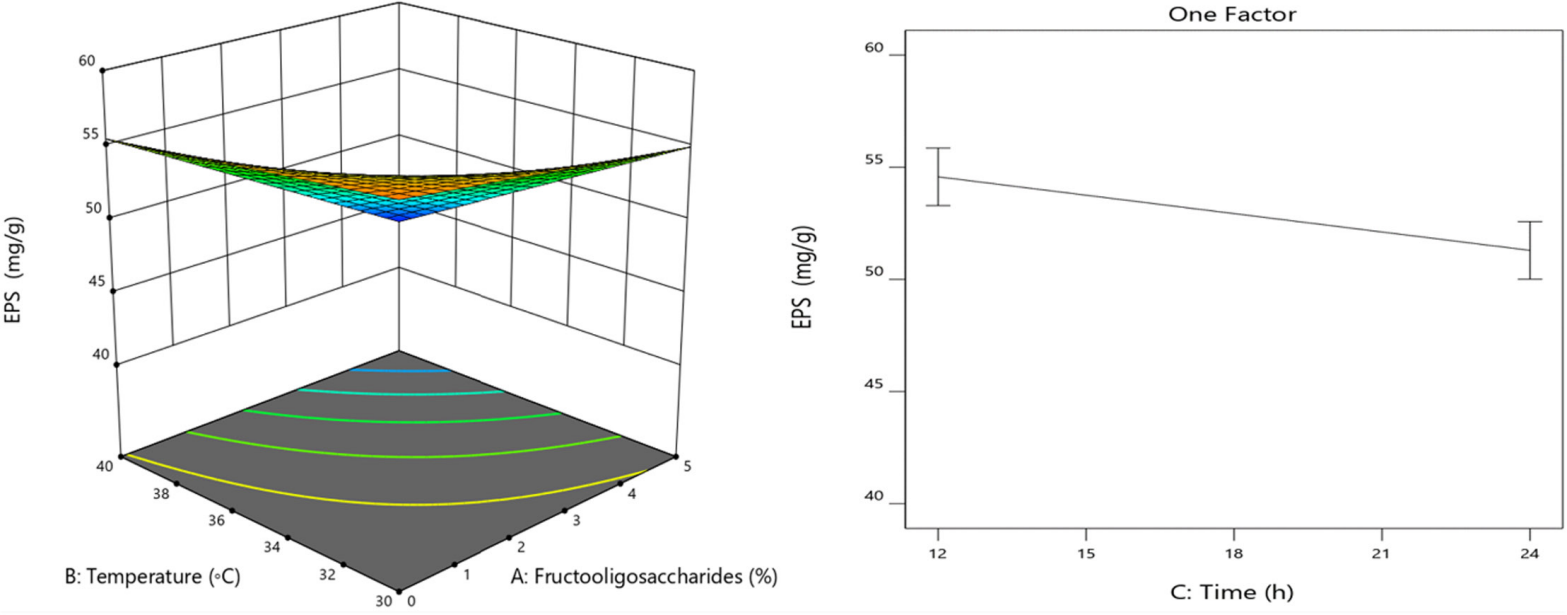
Interaction of FOS contents (%) with fermentation temperature (°C) and the effects of fermentation time (h) on EPS production.

According to Figure 2, the EPS content was reduced by increasing the fermentation temperature from 30 to 40°C and increasing the FOS content from 0% to 5%. The highest yield of EPS production was at 30°C, which is lower than the optimum growth temperature of bacteria. It has turned out that some mesophilic bacteria have higher yields and functions at temperatures below growth temperature (Degeest, Janssens, & De Vuyst, 2001; Degeest, Vaningelgem, & De Vuyst, 2001; Tallon et al., 2003). It seems that unfavorable growing conditions are favorable conditions for the production of EPSs by mesophilic bacteria because the sugar nucleotides, by cell walls, are used to produce EPSs. Studies have shown that the production performance of EPSs decreases after reaching a maximum, which is due to enzymes such as glycohydrolase produced by bacteria (Tsuda & Miyamoto, 2010).

The predicted model for EPSs gave the relationship between independent variables as follows:
(3)

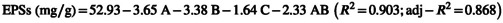




### 3.3 DPPH scavenging activity

**FIGURE 3:**
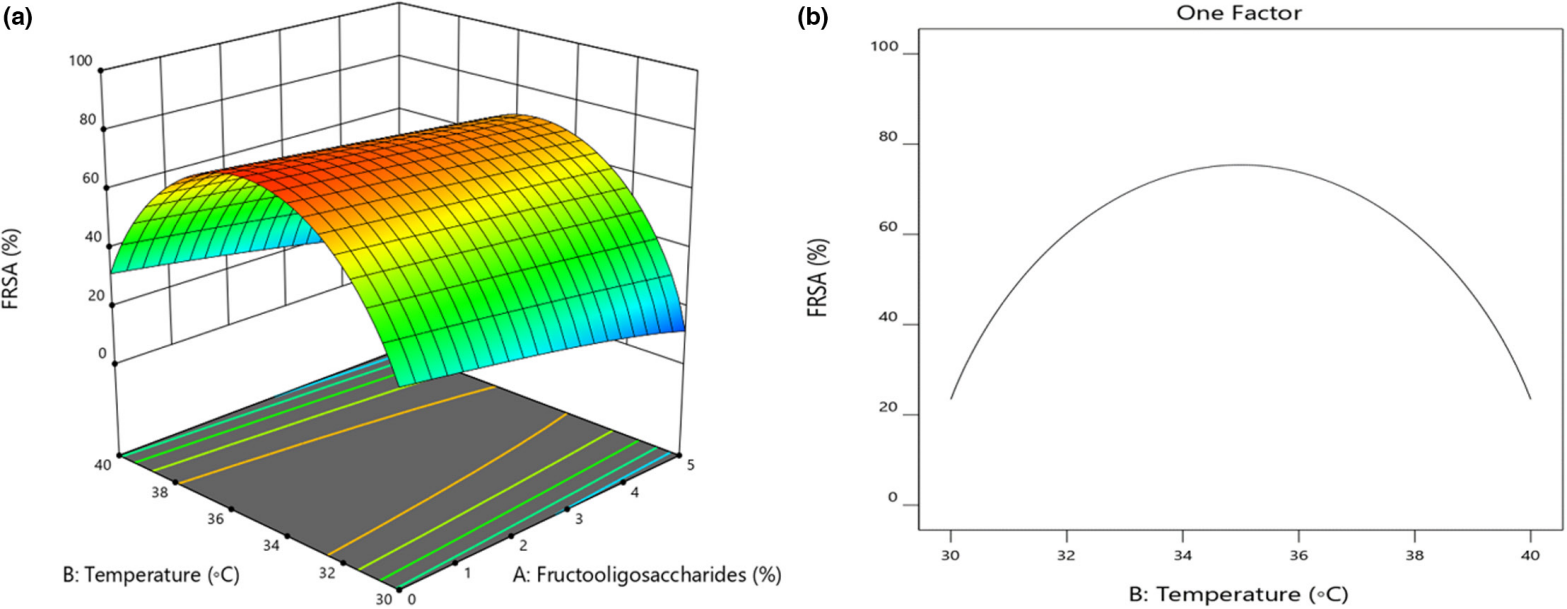
(a) Interaction of FOS contents (%) with fermentation temperature (°C) and (b) the effects of fermentation temperature on antioxidant activity.

According to the results, the effect of FOS content on antioxidant activity in sourdough was statistically significant (*p* < .05). According to Figure 3, which shows the interaction of FOS content from 0% to 5% and fermentation temperature from 30 to 35°C, antioxidant capacity increased with raising FOS and temperature. Sugar and inoculum can be enhanced to increase antioxidant activity. Increasing the sugar content and inoculum produces more biopeptides due to the activity of microorganisms, extracellular secretions of microorganisms, and more active phenolic compounds in the product, which increases the ability to inhibit DPPH free radicals (Kim et al., 2005). On the other, Liu et al. (2010) demonstrated that EPSs may act as electron donors and react with free radicals; hence, turn to more stable products to end free radical chain reactions. In return, the antioxidant activity decreased with increasing temperature (35–40°C). The results showed that at high temperatures and by increasing the FOS content, more biomass of *B. coagulans* was generated instead of producing organic acids. Hence, it has led to a decrease in the antioxidant activity, which was according to the results of Bomfim et al. (2020).

Predicted model (4) of free radical scavenging activity as a result of studied factors follows:

(4) $$\text{Free radical scavenging activity}\;\left(\text{FRSA}\right)\left(\%\right)=1149.47-107.29\;A-971.94\;B^{2}\;\left(R^{2}=0.979;\mathrm{adj}–R^{2}=0.976\right)$$

### 3.4 *B. coagulans* count

**FIGURE 4:**
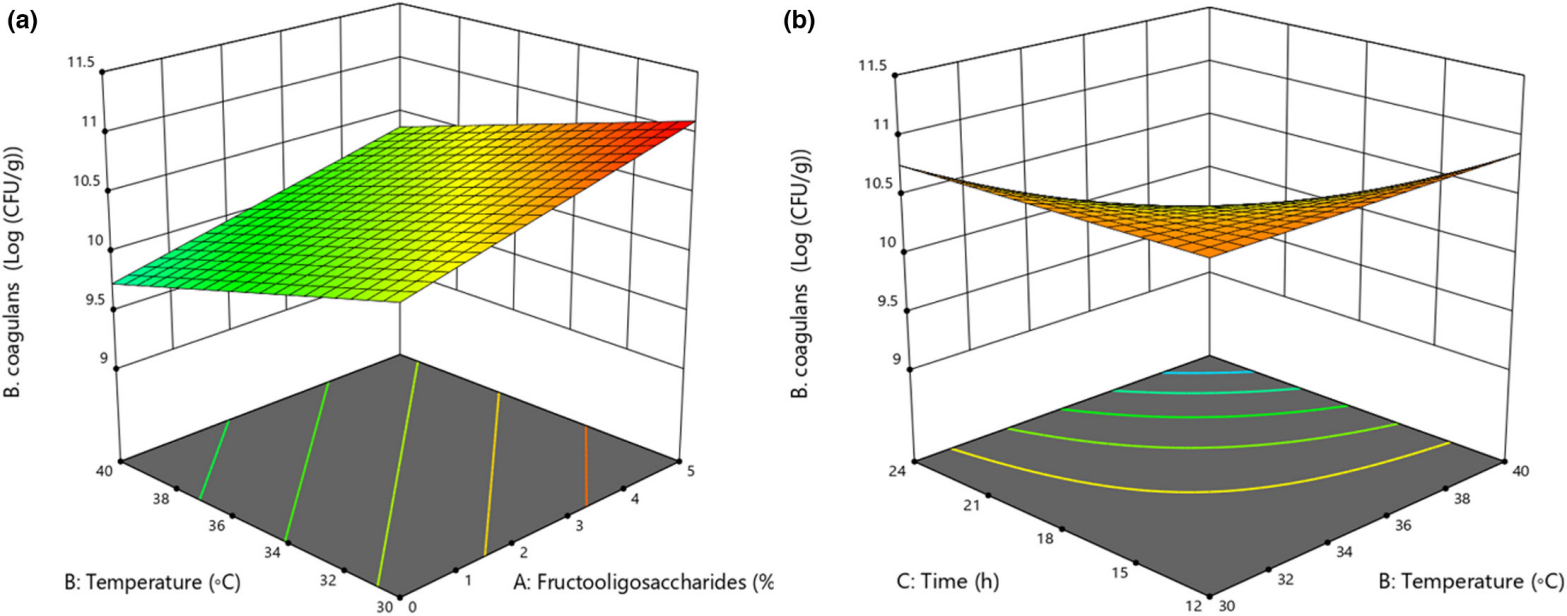
(a) Interaction of FOS contents (%) with fermentation temperature (°C), (b) fermentation temperature (°C) with fermentation time (h) on *B. coagulans* (Log (CFU/g)).

According to the results, the effect of fermentation time and temperature, and FOS content on the microbial growth of *B. coagulans* in the sourdough was statistically significant (*p* < .05). Furthermore, the interactions of FOS with fermentation temperature and further fermentation temperature with fermentation time significantly affected the growth of *B. coagulans* (Figure 4). By increasing FOS content (0%–5%) and decreasing the fermentation temperature (40–30°C), the *B. coagulans* count was increased. Prebiotics increased the growth and activity of the probiotic *B. coagulans*. Increasing the fermentation temperature (30–40°C) and time (8–24 h) led to an increase in the metabolic activity of *B. coagulans* cells and decreased their growth (Randazzo et al., 2013).

The predicted models for the number of *B. coagulans* include as follows:

(5) $$B.\;\text{coagulans}\;\text{count}\;\left(\mathrm{Log}\;\mathrm{CFU}/g\right)=10.42+0.3143\;A-0.3667\;B-0.4168\;C+0.4872\;\mathrm{AC}-0.389\;\mathrm{BC}\;\left(R^{2}=0.811;\mathrm{adj}–R^{2}=0.771\right)$$

### 3.5 Optimization of sourdough fermentation

In the current study, the fermentation conditions of sourdough production to obtain maximum EPSs and *B. coagulans* were optimized. Optimal conditions for maximum production of EPSs (59.284 mg/g) were predicted as FOS content of 0%, fermentation temperature of 30°C, and fermentation time of 12 h (with the desirability of 0.981) coded as sample A. Also, the optimal conditions for maximum growth of *B. coagulans* (11.179 Log CFU/g) were treatment with 4.706% FOS, fermentation temperature of 30°C, and fermentation time of 20 h with the desirability of 1 (coded as sample B). To validate the results of numerical optimization, two optimized samples (A and B) according to the predicted conditions for EPSs and *B. coagulans* count were prepared. Four other samples including C (sample A without *B*. *coagulans*), D (sample B without *B*. *coagulans* and FOS), E (sample B without FOS), and F (sample B without *B*. *coagulans*) as control samples of the optimized ones were also made (Table 2). All experiments were done in three replicates. As shown in Table 3, the experimental data are close to the predicted data (obtained via the models) confirming the validation of predicting models and optimization conditions.

**TABLE 3:** The results of the validation of the optimization.

Properties	Optimization of EPSs		Optimization of probiotic	
	Predicted	Actual	Predicted	Actual
pH	4.57^a^	4.48 ± 0.12^a^	4.75^a^	4.62 ± 0.13^a^
TTA (mL NaOH/g of sourdough)	11.18^a^	10.50 ± 1.02^a^	8.58^a^	7.9 ± 0.61^a^
FRSA (%)	32.06^a^	30.12 ± 2.08^a^	20.55^a^	18.73 ± 2.05^a^
EPSs (mg/g)	59.28^a^	59.70 ± 0.44^a^	54.47^a^	53.98 ± 0.95^a^
*B. coagulans* (log CFU/g)	11.00^a^	10.89 ± 0.19^a^	11.18^a^	11.20 ± 0.07^a^

Abbreviations: FRSA, free radical scavenging activity; actual data are average of three replicates; TTA, total titratable acidity. The same letter between predicted and actual columns for each sample shows no significant difference.

### 3.6 Microstructure of sourdoughs

**FIGURE 5:**
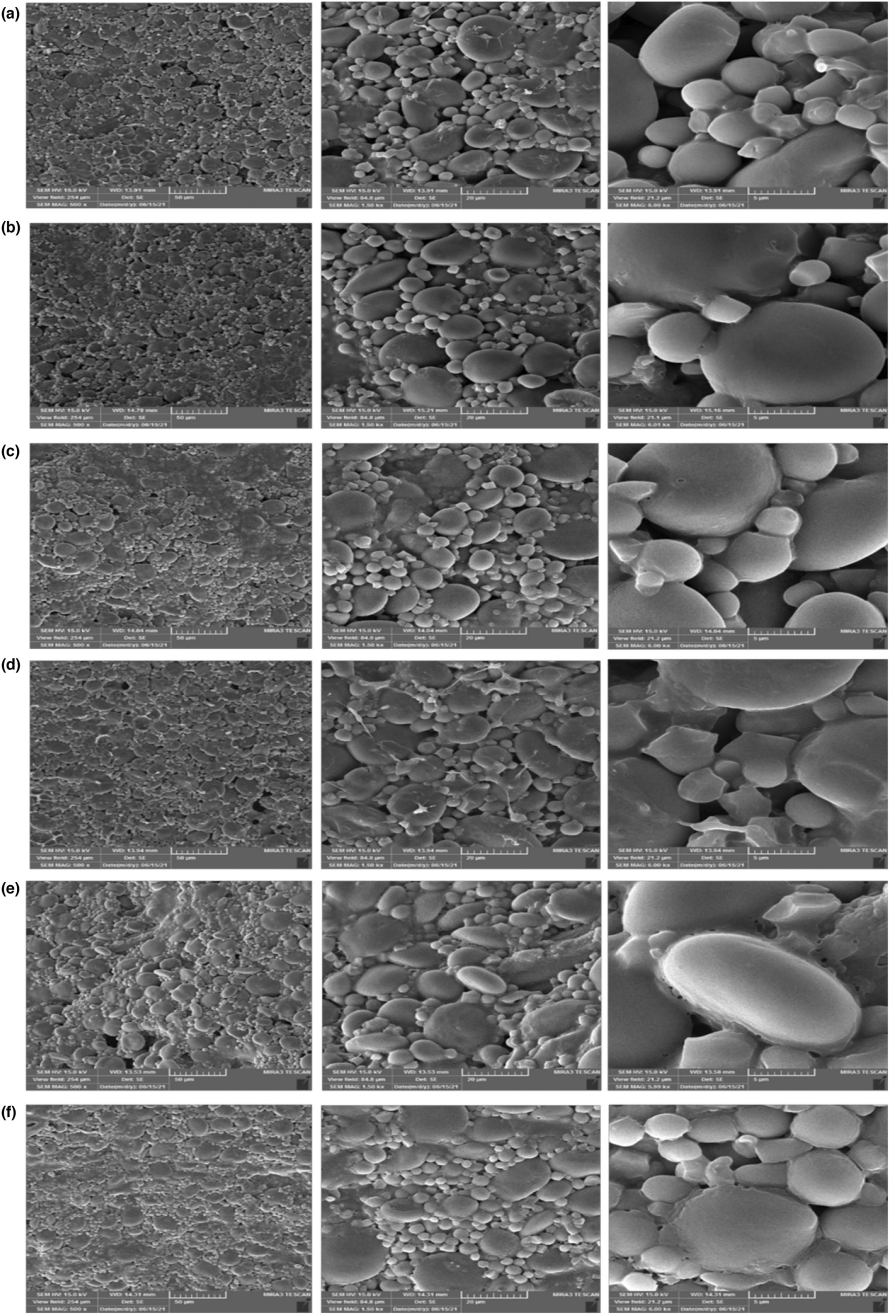
SEM micrographs of sourdoughs. a and b: Optimized samples; c: Control of a (without B.cogulans); d, e & f: Controls of sample b without FOS and B.coagulans, without B.coagulans, and without FOS, respectively.

The influence of FOS, *B. coagulans*, and produced EPSs on the microstructural properties of different sourdoughs were investigated by SEM microscopy (Figure 5). In the obtained images, most of the large and small starch granules had a smooth and uniform surface, and in the gluten–starch network, the starch grains were covered by a gluten matrix. Due to fermentation, the environment becomes more acidic and extricates (loses) the structure of gluten, which as a result, facilitates their interaction with starch granules (Siepmann et al., 2019). EPSs produced by *B. coagulans* and FOS in sourdough reduce the amount of water in starch due to the high tendency of oligosaccharides to bind to water and allow the sourdough to retain more moistness and increase its elasticity (Abedfar et al., 2019; İspirli et al., 2020; Park et al., 2016). FOS can prevent the accumulation of amylose and amylopectin as a physical hindrance (Salinas & Puppo, 2018). As a result, adding FOS and EPSs to the sourdough delays the retardation of amylopectin and improves the technological properties of the final product.

### 3.7 FTIR characterization

**FIGURE 6:**
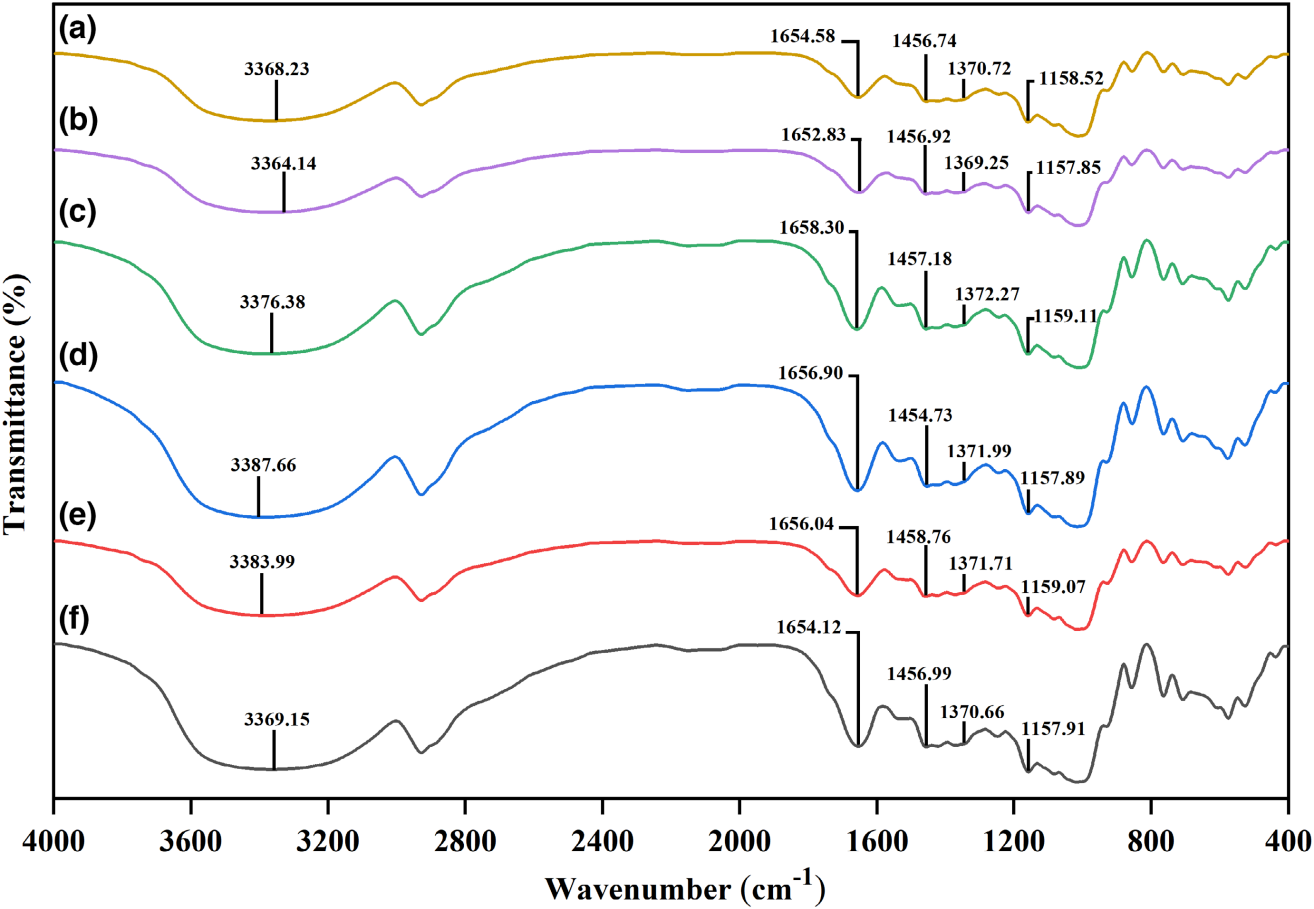
FTIR spectra of sourdoughs. a and b: Optimized samples; c: Control of a (without B.cogulans); d, e & f: Controls of sample b without FOS and B.coagulans, without B.coagulans, and without FOS, respectively.

FTIR was used to identify the functional groups and monitor the structural changes of the freeze‐dried sourdough samples of the frozen desiccant (Shen et al., 2013). The highest peak band generated in the region of approx. 3375 cm^−1^, which is related to the tensile vibrations of the hydroxyl group (‐OH), which indicates that the material is carbohydrates. Strong bands in the region of ~32,000, ~2900, ~1650, and ~1450 cm^−1^, respectively, to the tensile vibrations of hydroxyl groups, methylene groups (C‐H), the carbonyl group of amide I (C=O), and C‐H bending of protein and peptide amine and change the asymmetric forms of methyl (CH_3_) and methylene (CH_2_) proteins were assigned (Shen et al., 2013; Siepmann et al., 2019; Wang et al., 2010). The range of 1200–500 cm^−1^ is a fingerprint area for polysaccharides attributed to the tension of C‐C, and C‐O bonds with part of the C‐H bonds (Sujka et al., 2017; Siepmann et al., 2019). Against intuition, no significant new functional groups have emerged, and the chemical structure of the sourdough samples is similar (Figure 6).

### 3.8 DSC analysis

**FIGURE 7:**
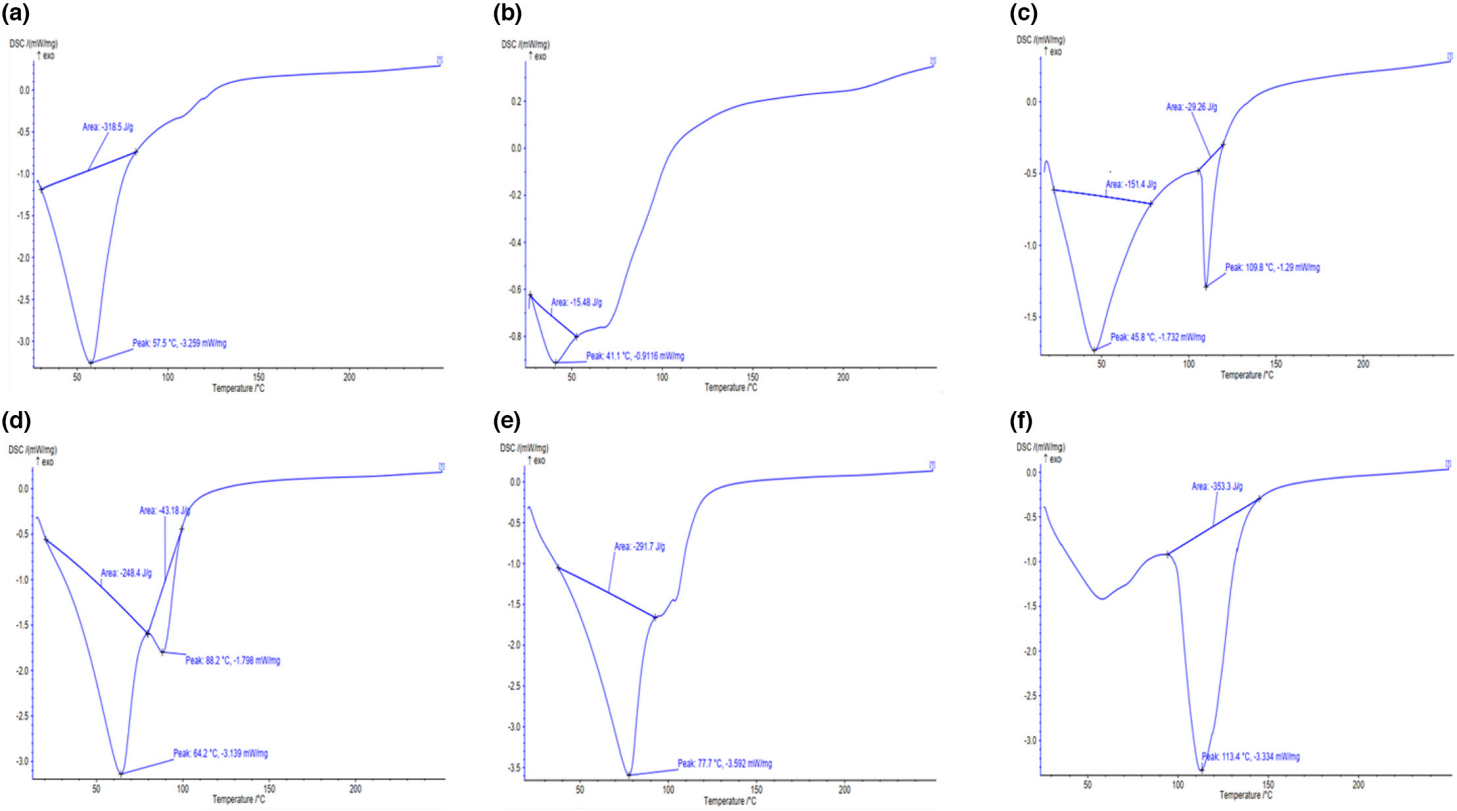
DSC thermograms of sourdoughs. a and b: Optimized samples; c: Control of A (without B.cogulans); d, e & f: Controls of sample b without FOS and B.coagulans, without B.coagulans, and without FOS, respectively.

The DSC method makes it possible to determine the thermal behavior of wheat starch during the partial baking process of sourdough using hermetic capsules. In the simulated baking process, the temperature of the fermented sourdoughs increased from 25 to 250°C. Figure 7 shows the experimental thermogram obtained by DSC. In DSC thermograms of sourdough samples, the recorded temperatures of wheat flour samples at **~**60 and **~**100°C were attributed to the starch gelatinization process and the appearance of amylose–lipid complexes, respectively (Corsetti et al., 1998). For sourdough without bacteria and FOS and the sample with FOS (C, D, and F), endothermic peaks were 46–64°C, and the sourdough with bacteria (A, B, and E) was 41–78°C. In addition, other endothermic peaks of 89–113°C were observed for nonbacterial and FOS sourdoughs (C and D) also sourdoughs containing FOS (F). In the present study, the absence of glass transfer temperature could be attributed to the production procedure, because the wheat gluten used in wheat flour did not leach (Nawrocka et al., 2017). Sourdoughs inoculated with *B. coagulans* (without FOS) showed higher enthalpy and displayed almost the same pattern. According to Δ*H*, *T*_p_ also increased in those samples. The high degree of crystallization, which provides structural stability (double helix length), leads to high transition temperatures. EPSs, like hydrocolloids, interact with gelatinized starch and amylose to make the starch structure more regular, thus causing to increase in the enthalpy of melting. These observations were consistent with the findings of Hadaegh et al. (2017). However, FOS may attach to the starch chain and complete amorphous regions before completing amylose from the granules. As a result, it increases starch gelatinization temperature and reduces retrograde amylopectin enthalpy (Aprodu et al., 2019). Therefore, samples containing bacteria and FOS had low peak temperatures and enthalpy. The microstructure of sourdoughs confirmed the results of the DSC analysis.

## 4 CONCLUSIONS

Due to the increasing consumption of bread worldwide and consumers' growing interest in functional foods, researchers are studying the utilization of probiotics and prebiotic compounds in bakery products to improve their quality and nutritional properties. Utilizing an ecosystem with high technological properties is essential for producing high‐quality bakery products. Based on the results of this study, there is the potential to improve the microbial and technological properties of type II sourdough by FOS and in situ‐produced EPSs by *B. coagulans*. The results showed that the inclusion of FOS and probiotics in the sourdough ecosystem caused a significant reduction in retrogradation starch and resulted in whole wheat sourdough with antistaling properties. The addition of FOS had a favorable effect on the increase of *B. coagulans* biomass and an adverse impact on the production of metabolites such as EPSs. The viable count of probiotics in sourdough declined over time. This sourdough is a potentially ideal culture medium for use in bakery products like bread due to its high microbial, functional, and nutritional properties.

## AUTHOR CONTRIBUTIONS

Zahra Farajinejad: Data curation (equal), formal analysis (equal), investigation (equal), writing‐original draft (lead). Forogh Mohtarami: Conceptualization (equal), methodology (equal), project administration (equal), supervision (equal), writing‐review and editing (equal). Mirkhalil Pirouzifard: Formal analysis (equal), investigation (equal), methodology (equal). Saber Amiri: Conceptualization (equal), investigation (equal), project administration (equal), data analysis, writing‐review and editing (equal). Hamed Hamishehkar: Conceptualization (equal), data curation (equal), formal analysis (equal), editing (equal).

## FUNDING INFORMATION

This research received no specific grant from any funding agency in the public, commercial, or not‐for‐profit sectors.

## CONFLICT OF INTEREST STATEMENT

The authors certify that they have no conflict of interest concerning this manuscript.

## Data Availability

Data will be made available on responsible request.
